# American Medical Association

**Published:** 1873-05

**Authors:** 


### AMERICAN MEDICAL ASSOCIATION.

The senior editor of this journal enjoyed the pleasure of an attendance upon the late session of the American Medical Association, at St. Louis. There were over five hundred members in attendance, and among them many of the best medical men of the country, and while other sessions of the body may have made equal or larger contributions to science, none have performed more important work for the permanency of the Association.

As is well known, it ran a most serious risk of being sundered at the meeting last year, in Philadelphia, but at the late session it might be said that ALL were agreed upon a reorganization which should place its future peace and safety beyond contingency. Besides the establishment of the "Judicial Council," which prevents for the future the introduction into the body for discussion of any exciting or disturbing question, initiatory steps were taken for changing the character of the membershlp (excluding representation from medical colleges, hospitals, etc.) so as to limit it to representatives from State Associations or local societies recognized as properly organized and deserving confidence as legitimate bodies by the State organizations. By these means the Association will be relieved of a large body of men who have no legitimate claim to membership, and of many exciting questions which have heretofore threatened its existence. In addition, a number of useless sections have been abolished; and the chairmen of those retained being required for the future to deliver an address before the Association upon some subject connected with the designated objects of the section, such places are not likely to be sought, as heretofore, by those who merely designed to get position and reputation, without rendering any return. The Association was most fortunate in the selection of the chairmen for the various sections. The distinguished veteran and father of the body, Dr S. N. Davis, of Chicago, was appointed Chairman of the Section upon Practical Medicine; Dr. S. D. Gross, of Philadelphia, the *greatest* of American surgeons, was appointed Chairman of the Section on Surgery; Dr. Theophilus Parvin, of Indianapolis, Ind., a most brilliant and accomplished medical gentleman, was appointed Chairman of the Section on Obstetrics and the Diseases of Women and Children; Dr. A. N. Talley, Professor of the Practice of Medicine in the University of South Carolina, at Columbia, a gentleman of most marked ability and the highest promise, was appointed Chairman of the Section on Medical Jurisprudence, Chemistry and Psychology; and lastly, Dr. A. N. Bell, of Brooklyn, New York, and the most eminent sanitarian of the United States, was appointed Chairman of the Section upon State Medicine and Public Hygeine. We have thus secured, most certainly, for the next meeting of the body, without providential interposition, *five* addresses, or reports, upon all the important branches of the profession, from some of the ablest men in the country.

And while the results of the late meeting were important, as the volume of Transactions will doubtless show, it was conceded upon all hands that *the great work* accomplished was in securing the harmony and perpetuity of the body and in preparing for future work.

The reception of the body by the profession and citizens of St. Louis was fully in accord with the dimensions and resources of the (at no distant day) second city of the Union and the grand country, of which it is the great emporium. Detroit, Michigan, was selected as the next place of meeting, and Dr. J. M. Toner, of Washington City, a most conspicuous and useful member of the Association and warmly devoted tc its interests, was unanimously elected President for the next year. We shall publish an abstract of the proceedings in the next number, for which we have no space in the present.

